# Educational Differences in Postmenopausal Breast Cancer – Quantifying Indirect Effects through Health Behaviors, Body Mass Index and Reproductive Patterns

**DOI:** 10.1371/journal.pone.0078690

**Published:** 2013-10-24

**Authors:** Ulla Arthur Hvidtfeldt, Theis Lange, Ingelise Andersen, Finn Diderichsen, Niels Keiding, Eva Prescott, Thorkild I. A. Sørensen, Anne Tjønneland, Naja Hulvej Rod

**Affiliations:** 1 Social Medicine Section, Department of Public Health, University of Copenhagen, Copenhagen, Denmark; 2 Section of Biostatistics, Department of Public Health, University of Copenhagen, Copenhagen, Denmark; 3 Institute of Cancer Epidemiology, Danish Cancer Society Research Center, Copenhagen, Denmark; 4 Department of Public Health and The Novo Nordisk Foundation Center for Basic Metabolic Research, Faculty of Health and Medical Sciences, University of Copenhagen, and Institute of Preventive Medicine, Bispebjerg and Frederiksberg Hospitals – part of the Copenhagen University Hospital, The Capital Region, Copenhagen, Denmark; 5 Department of Cardiology, Bispebjerg University Hospital, Copenhagen, Denmark; 6 The Copenhagen City Heart Study, Bispebjerg University Hospital, Copenhagen, Denmark; Kyushu University Faculty of Medical Science, Japan

## Abstract

Studying mechanisms underlying social inequality in postmenopausal breast cancer is important in order to develop prevention strategies. Standard methods for investigating indirect effects, by comparing crude models to adjusted, are often biased. We applied a new method enabling the decomposition of the effect of educational level on breast cancer incidence into indirect effects through reproductive patterns (parity and age at first birth), body mass index and health behavior (alcohol consumption, physical inactivity, and hormone therapy use). The study was based on a pooled cohort of 6 studies from the Copenhagen area including 33,562 women (1,733 breast cancer cases) aged 50–70 years at baseline. The crude absolute rate of breast cancer was 399 cases per 100,000 person-years. A high educational level compared to low was associated with 74 (95% CI 22–125) extra breast cancer cases per 100,000 person-years at risk. Of these, 26% (95% CI 14%–69%) could be attributed to alcohol consumption. Similar effects were observed for age at first birth (32%; 95% CI 10%–257%), parity (19%; 95%CI 10%–45%), and hormone therapy use (10%; 95% CI 6%–18%). Educational level modified the effect of physical activity on breast cancer. In conclusion, this analysis suggests that a substantial number of the excess postmenopausal breast cancer events among women with a high educational level compared to a low can be attributed to differences in alcohol consumption, use of hormone therapy, and reproductive patterns. Women of high educational level may be more vulnerable to physical inactivity compared to women of low educational level.

## Introduction

Breast cancer is the most common cancer among women worldwide [1], and the main cause of early death among women in developed countries [2]. Unlike most other types of cancer, breast cancer is more frequently observed in women of higher socioeconomic position (SEP) [3-7]. The pathways through which SEP affects the risk of breast cancer are not well understood; yet strategies towards reducing social inequalities in cancer rely on a greater understanding of which risk factors mediate the effect of SEP on cancer incidence.

Several studies support an etiological role of sex-steroid hormones in the development of postmenopausal breast cancer [8-10], and reproductive factors have been found to affect the risk [11,12]. Women of higher SEP generally have fewer babies and give birth at older ages [12]. But adverse effects have also been documented for modifiable health behaviors that may affect hormone levels, such as alcohol consumption, physical inactivity, and hormone therapy (HT) use as well as high body mass index (BMI) [13-17]. Women with higher SEP tend to drink more alcohol which may also affect the risk of breast cancer for this group [18].

In line with this, previous studies have focused on indirect effects in social inequality in breast cancer [18-22]. A recent study found a substantial reduction in the social inequality in breast cancer after adjustment for reproductive factors, and adding health behaviors to the model further decreased this association [18]. However, indirect effects derived by comparing multiplicative models with and without the potential mediator are potentially biased [23,24]. For instance, these methods cannot account for situations where exposure and mediator interact in their effect on the outcome. Furthermore, even in the absence of confounding, the total effect of an exposure measured by a ratio (hazard, odds etc.) is not generally decomposable into indirect effects (ratios will change after adjustment even if there is no true effect of the exposure on the mediator) using simple regression techniques.

The objective of this study was to investigate the underlying mechanisms linking educational level to breast cancer by quantifying the indirect effects through health behaviors, BMI and reproductive patterns. We apply a newly developed method for mediation analysis [25], which quantifies the number of breast cancer cases that can be ascribed to each mediating factor, thereby improving the understanding of social inequality in breast cancer.

## Methods

### Study population

The analyses were based on the Social Inequality in Cancer (SIC) database derived by pooling several large independent population studies from the Copenhagen area: The Diet, Cancer, and Health Study (DCHS) [26], The Copenhagen City Heart Study (CCHS) [27], and four selected studies from the Cohorts at the Research Centre for Prevention and Health (CRCPH) [28]: Monica I-III and Inter-99. All participants filled in a self-administered questionnaire on health status, health behaviors, and reproductive factors at baseline and this information was harmonized across the studies. We included postmenopausal women defined as women aged 50+ years at baseline. The baseline characteristics of the included studies are described in Table 1. We excluded women with a history of cancer (other than non-melanoma skin cancer) and women born before 1921 since the central registries do not contain information on education for these birth cohorts. In total, the study included 33 562 women.

**Table 1:** Baseline Characteristics of Included Studies, Copenhagen, Denmark, 1981-2001.

			**Baseline cohort^a^**		**Year of questionnaire**		**Mean age, years (range)**		**Person-years of follow-up**		**BC events**		**High educational level^b^**
DCH			28 654		1993-97		56.2 (50-65)		347 320		1397		19.3%
CCHS II			2265		1981-83		55.7 (50-62)		47 327		190		7.6%
CRCPH													
	Monica 1		840		1982-84		54.5 (50-60)		17 870		66		7.5%
	Monica 2		265		1986-87		55.5 (50-60)		5004		26		8.3%
	Monica 3		555		1991-92		59.8 (50-70)		8155		30		7.8%
	Inter99		983		1999-2001		52.8 (50-60)		8583		24		21.5%
**Total**			**33 562**		**1981-2001**		**56.1**		**434 260**		**1733**		**18,0%**

Abbreviations: BC events, breast cancer events; CCHS, Copenhagen City Heart Study (2^nd^ wave); CRCPH, The Cohorts at the Research

Centre for Prevention and Health; DCH, Diet, Cancer, and Health Study.

^a^ Sample size after exclusion of participants with baseline cancer and missing information on educational level, body mass index, alcohol consumption and parity.

^b^ ’High’ educational level defined as ≥ 15 years of education.

### Measures of SEP

The SIC-database was linked to sociodemographic information from Statistics Denmark from 1980 and onwards. SEP was defined as highest attained educational level one year before baseline and categorized as: “low” (8–11 years of basic schooling), “medium” (11–14 years; upper secondary or vocational training), and “high” (15+ years) education. Educational level was chosen over income or occupation because it is a more constant measure of lifelong social status [6,29].

### Measures of health behavior

Alcohol was assessed as consumption of beer, wine, and spirits in response categories of “never/almost never”, “monthly”, “weekly”, and “daily” as well as the average number of drinks per week within these categories. We categorized the total intake in groups of <1, 1–7, and >7 drinks/week. Leisure time physical activity was assessed in a similar manner across studies: In the DCH study, the average number of hours spent in the past year on various types of activity (e.g. cycling, walking) was assessed along with number of hours becoming sweaty or short of breath as a result of these activities. Similarly, the CCHS and the CRCPH assessed the weekly level of physical activity during the past year in four categories ranging from being almost entirely inactive to engaging in vigorous physical activity. As very few participants (7.5 %) reported being highly physical active the measures were harmonized to a 3-level variable ranging from sedentary (<2 hours of light physical activity) to active (>4 hours of light activity or >2 hours of vigorous activity per week). Parity was classified into four groups ranging from 0 to 3+ children. Age at first birth was grouped as <25 years, 25–29 years, 30+ years, and nulliparous. Current HT use was classified as yes vs. no.

#### Body mass index

Only 1.4% of the women were underweight (BMI < 18.5 kg/m^2^), and thus BMI was categorized as normal weight (<25 kg/m^2^), overweight (25–29.9 kg/m^2^), and obese (30+ kg/m^2^).

### Follow-up

The end-point was defined as first incidence of breast cancer. Time and cause of disease were obtained from The Danish Cancer Registry which is based on ICD 7 and ICD 10 codes (ICD7 code 174 and ICD10 code C50) [30]. Information on emigration and deaths was obtained from the Registry for Population Statistics and Statistics Denmark. Participants were followed from baseline to date of breast cancer event, date of death, emigration or end of follow-up (December 31, 2009), whichever occurred first.

### Ethics statement

The study was approved by the Danish Data Protection Agency. All participants signed written consent before participating.

### Statistical methods

The individual studies were pooled [31,32], and models were stratified according to age at baseline and study to account for period effects. The stratification according to study origin also accounted for differences in questionnaire design. We assessed mediation by computing natural direct and indirect effects as originally proposed by Robins and Greenland [33] and Pearl [34]. Natural direct effects are defined as the change in outcome that would be observed if the exposure could be changed (e.g. *high* to *low*), but leaving the mediators unchanged (corresponding to *high* exposure). Likewise, natural indirect (i.e. mediated) effects are defined as the change in outcome when exposure is kept fixed, but the mediator is changed to the value it would take if exposure was changed. Thus, natural indirect effects can be thought of as the effect of SEP mediated through a specific factor. Natural direct and indirect effects were directly parameterized following the method of Lange et al [25]. A step-by-step description of the procedure is provided in Web Appendix S1. Effects were assessed on the additive hazard scale using additive hazard models as the natural effects model. Covariates were tested for time-dependent effects [35]. Mediated proportions were computed as the indirect effect divided by the total effects and 95% confidence intervals were derived by simulation based on the variance and covariance of the direct and indirect effects. We tested for interaction between SEP and each of the mediators by a Wald test, and interactions were presented as mediated interactive effects [36]. 

**Figure 1:**
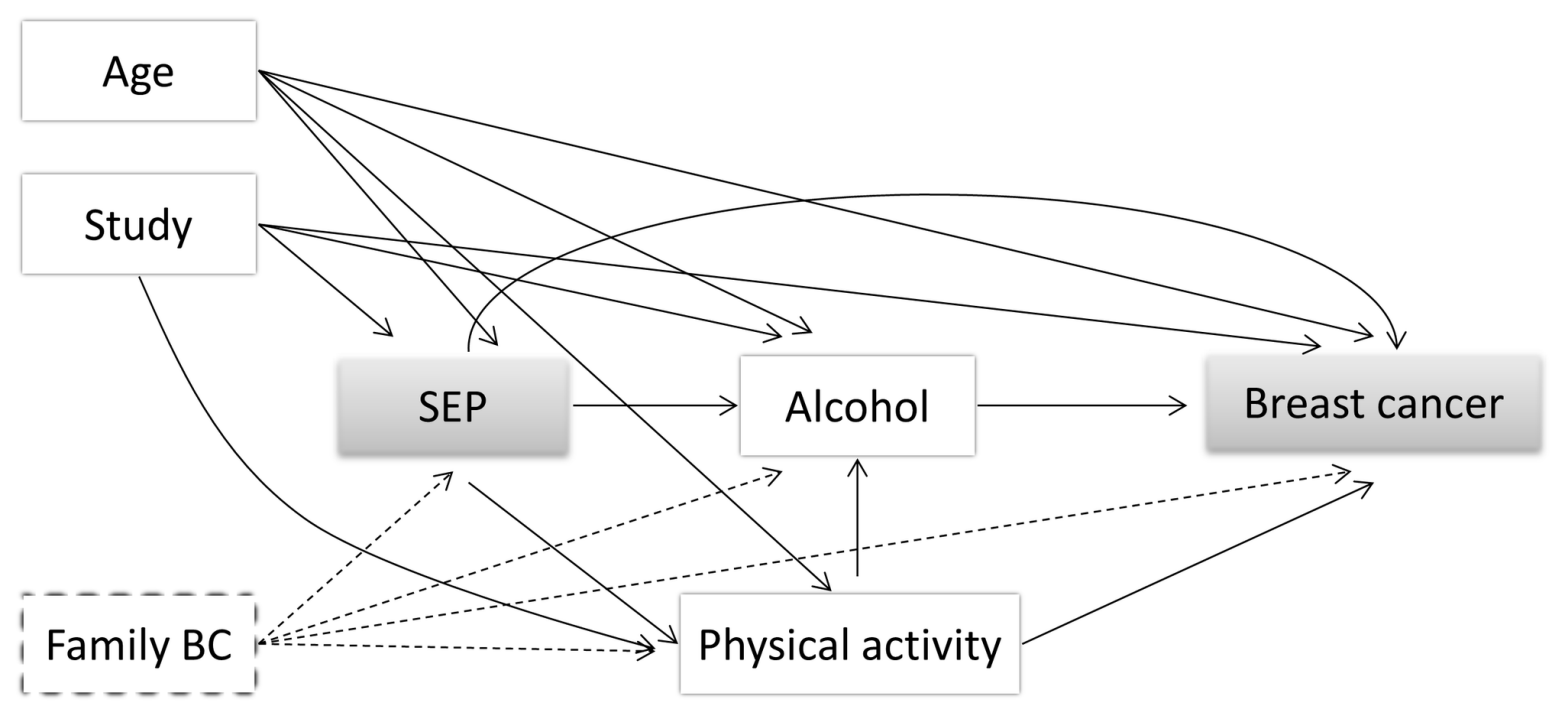
Model describing intertwined pathways, exemplified by the effect of SEP on breast cancer through alcohol consumption and physical activity. Arrows represent causal pathways and dotted lines represent unmeasured factors.

The analyses include multiple potential mediators. For each potential mediator both the effect mediated through that variable and the effect through other causal pathways are estimated. It must be stressed that the analysis only includes one mediator at a time; thus the direct effect associated with one mediator can (partly) constitute the indirect effect of another, i.e. the effects are intertwined (Figure 1). To address this issue, we assessed mediation through a variable combined by the individual mediators showing significant mediating effects (alcohol consumption, HT use and reproduction). If the effect mediated through the combined variable is equal to the sum of the individual indirect effects this indicates that the individual mediators represent distinct non-intertwined causal pathways.

Sensitivity analyses included: 1) Assessing heterogeneity between study-specific effects by including an interaction term between educational level and study. Also, pooled estimates were compared by systematically removing each individual study at a time to confirm that no single study strongly influenced the pooled estimates; 2) excluding the first 3 years after baseline to rule out reverse causality; 3) assessing the association between educational level and breast cancer stratified by birth cohort (i.e. women born ≤ 1939 or after) to account for the fact that younger birth cohorts generally have a higher educational level and the shortest follow-up time; and 4) Assessing indirect effects of other anthropometric measures of adiposity: waist-to-hip-ratio and waist circumference adjusted for BMI (N=30 453) [37,38].

## Results

In total, the pooled cohort comprised 33 562 women aged 50 to 70 years at baseline who experienced 1733 breast cancer events during 434 260 years of follow-up (Table 1). The percentage of women with a high educational level varied from 7.5 to 21.5 between the cohorts. These women were generally less obese, had a higher alcohol intake and physical activity level, had fewer children and higher age at first birth, and were more likely current users of HT compared to women with a low educational level (Table 2). 

**Table 2:** Characteristics of participants according to educational level, Copenhagen, Denmark, 1981-2001.

			**Educational level, %**					
**Variable**			**Low**		**Medium**		**High**	P*
N			12 273		15 257		6032	
BMI, kg/m^2^								*<0.01*
	*< 25*		46.2		53.1		62.0	
	*25-29*		35.6		34.2		28.7	
	*30+*		18.3		12.7		9.3	
Alcohol consumption, drinks/wk								*<0.01*
	*<1*		26.3		14.9		9.9	
	*1-7*		44.4		46.2		42.5	
	*7+*		29.4		38.9		47.5	
Physical activity,								*<0.01*
	*Inactive*		19.5		17.0		16.0	
	*Low*		41.9		42.4		39.2	
	*Moderate/high*		38.5		40.6		44.8	
Parity								*<0.01*
	*0*		10.3		11.8		16.3	
	*1*		15.5		17.0		15.9	
	*2*		40.9		48.5		43.0	
	*3+*		33.3		22.7		24.9	
Age at first birth^a^								*<0.01*
	*< 25 years*		66.4		53.1		33.6	
	*25-29 years*		16.7		26.6		36.0	
	*30+ years*		4.6		7.5		13.5	
	*Nulliparous*		12.4		12.8		16.9	
Current HT use^b^								*<0.01*
	*Yes*		28.5		30.2		31.0	

Abbreviations: BMI, body mass index; HT, hormone therapy.

“Low”= 8-11 years of basic schooling; ”Medium”= 11-14 years (upper secondary or vocational education);

”High” ≥ 15 years of education.

^*^ *P*-value (2 sided) for comparing mediators to educational levels were obtained from Pearson χ^2^-test.

^a^ Excluding participants with missing information on age at first birth, N=3475.

^b^ Excluding participants with missing information on HT use, N=2641.

The crude absolute rate of breast cancer in this cohort was 399 cases per 100 000 person-years at risk. The total effect of educational level and the effect of each mediator on breast cancer are presented in Table 3. The total effect is interpreted as the extra number of cases that could be prevented by hypothetically changing all the women’s educational level from e.g. *high* to *low* under the assumption that the observed associations provide reasonable estimates of causal effects. The effect of a *medium* educational level was similar to that of women with a *high*, that is 74 (95% CI = 22–125) extra cases per 100 000 person-years at risk compared to women with a *low* educational level. An alcohol consumption of 7+ drinks/week was associated with 123 (95% CI = 69–178) extra cases compared to abstinence. Being nulliparous compared to having 3+ children was associated with 180 (95% CI = 108–251) and 155 (95% CI = 80–230) extra cases compared to women giving birth before the age of 25 years. Being a current user of HT was associated with 270 (95% CI = 222–318) extra cases compared to current non-users. We did not observe an association between breast cancer and BMI or physical activity.

**Table 3:** Total effects of educational level and effects of each potential mediator on breast cancer, Copenhagen, Denmark, 1981-2001.

			**Additional cases per 100 000 py**	**95% CI**
Educational level^a^				
	*Low -> Medium*		70	29, 112
	*Low -> High*		74	22, 125
BMI^b^, kg/m^2^				
	*< 25 -> 25-29*		-19	-60, 23
	*< 25 -> 30+*		-2	-59, 55
Alcohol consumption^b^, drinks/wk				
	*<1 -> 1-7*		26	-22, 74
	*<1 -> 7+*		123	69, 178
Physical activity^b^				
	*Moderate/high -> low*		2	-39, 43
	*Moderate/high -> inactive*		1	-54, 55
Parity^b^				
	*3+ -> 2*		60	16, 104
	*3+ -> 1*		102	42, 163
	*3+ -> 0*		180	108, 251
Age at first birth^b,c^, years				
	*< 25 -> 25-29*		24	-25, 73
	*< 25 -> 30+*		62	-20, 144
	*< 25 -> nulliparous*		155	80, 230
Current HT use^b^,^d^				
	*No -> Yes*		270	222, 318

Abbreviations: BMI, body mass index; HT, hormone therapy; PY, person-years.

“Low”= 8-11 years of basic schooling; ”Medium”= 11-14 years

(upper secondary or vocational education); ”High” ≥ 15 years of education.

^a^ Adjusted for age and study.

^b^ Adjusted for SEP, age and study.

^c^ Excluding participants with missing information on age at first birth, N=3475.

^d^ Excluding participants with missing information on HT use, N=2641.

Direct and indirect effects as well as mediated proportions of each mediator on breast cancer risk are presented in Table 4. The indirect effect of e.g. alcohol is the number of cases that could be prevented if the consumption of women with a *high* educational level was changed to those of *low*. Correspondingly, the direct effect is the effect associated with a change in the women’s educational level – but assuming their alcohol consumption remained unchanged. The indirect effect of alcohol consumption for *high* educational level compared to *low* was 19 (95% CI = 11–27) additional cases per 100 000 person-years at risk corresponding to a mediated proportion of 26% (95% CI = 14%–69%). The effect mediated through parity was 14 (95% CI = 8–19) additional cases corresponding to 19% (95% CI = 10%–45%). The corresponding mediated proportion for age at first birth was 32% (95% CI = 10%–257%) and 10% (95% CI = 6%–18%) for current HT use. We observed an interaction between SEP and physical activity (*P* for interaction = 0.01) with an indirect effect through physical activity of 2 (95% CI = -1–5) for *high* compared to *low* educational level, and a mediated interactive effect of -10 (95% CI = -16 - -4). This means that women of low educational level may be less vulnerable to physical inactivity than women of high educational level.

**Table 4:** Direct and Mediated Effects per 100 000 person-years of Educational Level on Breast Cancer for each Mediator derived from Multinominal Logistic Regression Parameter Estimates and the Additive Hazards Model, Copenhagen, Denmark, 1981-2001.

**Potential mediators^a^**	**Educational level**	**Direct effect^b^ (additional cases per 100 000 py)**	**95% CI**	**Mediated effect^b^ (additional cases per 100 000 py)**	**95% CI**	**Mediated proportion**	**95% CI**
*BMI*	Low -> Medium	66	24, 108	0	-4, 4	0%	-7%, 6%
	Low -> High	65	13, 118	1	-6, 8	1%	-24%, 14%
*Alcohol consumption*	Low -> Medium	59	16, 101	10	5, 15	15%	-1%, 28%
	Low -> High	55	3, 107	19	11, 27	26%	14%, 69%
*Parity*	Low -> Medium	61	19, 103	10	6, 14	14%	8%, 36%
	Low -> High	60	9, 111	14	8, 19	19%	10%, 45%
*Age at first birth^c^*	Low -> Medium	54	7, 102	7	1, 14	12%	0%, 21%
	Low -> High	39	-20, 99	19	4, 33	32%	10%, 257%
*HT use^d^*	Low -> Medium	67	24, 110	5	4, 6	7%	4%, 19%
	Low -> High	63	8, 119	7	6, 8	10%	6%, 18%

Abbreviations: BMI, body mass index; CI, confidence interval; HT, hormone therapy; PY, person-years.

^a^ In categories of: BMI <25 kg/m^2^, 25-29.9 kg/m^2^, and 30+ kg/m^2^; Alcohol <1, 1-7, and >7 drinks/week; Physical activity <2 hours of light physical activity; 2-4 hours of light physical activity, and >4 hours of light activity/> 2 hours of vigorous activity per week; Parity 0, 1, 2, 3+; Age at first birth <25, 25-29, 30+ yrs, and nulliparous. HT use ‘yes’ vs. ‘no’.

^b^ Adjusted for age and study.

^c^ Excluding participants with missing information on age at first birth, N=3475.

^d^ Excluding participants with missing information on HT use, N=2641.

In additional analyses we combined the factors showing indirect effects into a variable with 8 levels corresponding to all unique combinations of the three (binary) factors (≤7 vs. >7 drinks/wk), reproduction (<30 years at first birth vs. ≥ 30/nulliparous) and HT use. The total effect for *high* compared to *low* educational level was 69 (95% CI = 11–126) additional breast cancer cases where 39 (95% CI = 28–51), or 57% (95% CI = 33%–183%), could be attributed to the factors combined. 

Analyses of the heterogeneity between study-specific effects confirmed that the pooled estimates can be considered appropriate summaries of the study-specific data (*P* for interaction = 0.50). We also performed four sensitivity analyses of which the first showed that removing one individual study at a time did not affect the risk estimates. In the second analysis, we found that excluding cases occurring within the first 3 years after baseline also did not affect the risk estimates. Thirdly, we did not observe evidence of differential effects of educational level on breast cancer according to birth cohort (*P* for interaction = 0.81). In women born before 1940, 77 (95% CI = 21–172) extra cases per 100 000 person-years at risk were observed for women with a *medium* vs. *low* educational level. The corresponding number of additional cases was 63 (95% CI = -14–139) for women with a *high* educational level compared to a *low*. In women born after 1940, the number of additional cases was 62 (95% CI = -0.4–124) for women with a *medium* vs. *low* educational level and 86 (95% CI = 10–162) for women with a *high* educational level. Finally, the subanalysis investigating the indirect effect of waist-to-hip ratio and waist circumference adjusted for BMI did not show results markedly different from those of BMI (data not shown).

## Discussion

We applied a new method for assessing mediation by estimating natural direct and indirect effects based on the Aalen additive hazards model. Our findings suggest that approximately 26% of the excess breast cancer cases among the highly educated women could be prevented if they changed their alcohol consumption corresponding to the women of low educational level. A similar proportion of cases could be prevented if women of high educational level had the same reproductive patterns as women of low educational level. It also appeared that women of high educational level were more vulnerable to physical inactivity compared to women of low educational level.

 Our findings are qualitatively in agreement with previous studies [18-22,39]. A recent large pooled analysis found a substantial reduction in incidence after adjustment for reproductive factors and additional adjustment for known lifestyle risk factors removed the remaining social inequality [18]. Also, a large Norwegian study examined the association of reproductive and lifestyle factors in stepwise statistical analyses and concluded that 26% and 23% of the social inequality in breast cancer was explained by parity and alcohol consumption, respectively [21]. These studies, however, were based on comparing relative measures before and after adjustment for potential mediators and thus may be biased due to possible exposure-mediator interaction and non-linearity [23,24]. The present study has added to the literature by quantifying the additional number of breast cancer cases that can be ascribed to each potential mediator in a mathematically consistent manner and documented that the previously observed evidence for mediation was not severely affected by the potential biases of the employed methods.

The causal link between SEP and breast cancer through the investigated factors is biologically plausible as alcohol consumption and reproductive behavior may affect endogenous sex-hormone levels [40,41]. However, non-causal explanations may also apply. For instance, a population-based breast cancer screening program offering biennial screenings to women aged 50-69 years was introduced in Copenhagen in 1991. If women of high educational level attended screening visits to a higher degree than women of low educational level, the detection rate of cases for the two groups is skewed. Data on screening participation were not available for the present study. A previous study on the Danish screening program, however, found a U-shaped relation between education and non-participation in breast cancer screening, indicating that this may not be the main explanation for the observed higher incidence in women of higher educational level [42]. 

The interpretation of our study was strengthened by the large sample size, the prospective design, the linkage to population-based registers on disease, death and emigration, and the application of a new method for mediation analysis especially developed to address our research question. However, several limitations should also be considered. Women of high educational level were overrepresented in the cohort which may raise concerns about the generalizability of the results. The study included 6 cohorts to obtain more statistical power and the harmonization of variables may have compromised the precision of the measured mediators. Also, changes in health behaviors during follow-up are not captured by the baseline measurements. For instance, HT use may have changed over time, especially considering the health concerns following the initial release of results from the Women’s Health Initiative trial in 2002 [43]. As mentioned previously, non-differential misclassification (i.e. measurement error) on the mediator generally leads to underestimates of mediating effects [44], which may partly explain the modest indirect effects of some mediators observed in our study. Also, the analyses suggested that the indirect effect of physical inactivity varied by educational level with the strongest indirect effect observed for those with a high educational level. This may imply that women of high educational level are more vulnerable to physical inactivity compared to women of low educational level. However, the observed interaction could also be due to the relatively crude categorization of physical activity which may have resulted in some degree of differential misclassification of physical activity level across educational groups. In addition, the broad baseline period (1980-2001) raises questions of study heterogeneity as the educational level of women has increased markedly within the study period combined with the fact that participation rates have declined (70% participation in CCHS versus 37% in the DCH study). This leads to overrepresentation of highly educated women in surveys with a later baseline, and combining these cohorts may have attenuated the overall effect estimates of SEP on breast cancer as implications of belonging to a specific social class may vary over time [7]. We did, however, examine the association of educational level and breast cancer stratified by birth cohort and did not find evidence of differential effects. In addition, the model assumes that there are no unmeasured confounders of the investigated relations. While the database includes a great number of measured covariates, information on family history of breast cancer was not available. It is likely that previous cancers in the family may have affected participant’s SEP, health behavior and their own risk of breast cancer (cf. Figure 1). However, the confounding effect is likely to be small as the population attributable fraction of family history to breast cancer is modest [45]. This study analyzed indirect effects for invasive breast cancers as a single disease; however, it is now widely accepted that different subtypes of breast tumors, such as estrogen receptor positive versus negative, may have distinct etiologies [46,47]. Thus, future studies should address indirect effects of these factors according to breast cancer subtypes. In the present study multiple potential mediators were assessed. As mentioned previously, the analysis only included one mediator at a time; thus the direct effect associated with one mediator can (partly) constitute the indirect effect of another mediator. A logical question is if the different indirect effects can be added together and the remaining direct effect in this way reduced. Analyzing mediation through multiple mediators is an active area of research, but powerful techniques are still lacking. However, summation of indirect effects is only meaningful if the different mediators represent distinct non-intertwined causal pathways and if the effects are all linear (i.e. no interactions). Intertwined pathways involve exposure-dependent confounding of the relations, which makes it less straight forward to associate specific indirect effects to the different mediators. Thus, the indirect path through for instance alcohol consumption may be affected by other behavioral factors (cf. Figure 1), and the effect of the other investigated mediators may also likely be intertwined. However, we did not observe an effect of BMI on breast cancer and the effect of physical activity was small, which indicates that bias through these factors may be negligible in our data. Combining the three factors with substantial indirect effects indicated that the effects were somewhat intertwined since the combined effect did not reach the sum of the separate indirect effects. Dichotomizing the three variables introduces further misclassification to the mediator, and the measured indirect effect through the combined variable may thus be underestimated. 

In conclusion, our findings indicate that the higher incidence of postmenopausal breast cancer among women of high educational level is primarily mediated through alcohol consumption, reproductive behaviors and HT use. Thus, the social inequality in breast cancer would in theory possibly be reduced, if women with a higher educational level could be motivated to give birth to more children at younger ages and reduced their alcohol intake and HT use corresponding to the levels of women with a lower educational level. Future studies should confirm the findings of the present study and address other pathways as well as distinct subtypes of breast cancer, and techniques for quantifying mediating effects of several factors simultaneously need further attention.

## Supporting Information

Appendix S1 (DOCX)
