# Supplementary material for: Educational Differences in Postmenopausal Breast Cancer – Quantifying Indirect Effects through Health Behaviors, Body Mass Index and Reproductive Patterns
Source: PLoS One. 2013 Oct 24;8(10):e78690. doi: 10.1371/journal.pone.0078690 (PMC3812044; doi:10.1371/journal.pone.0078690)
Supplement: Appendix S1 — (DOCX) [file pone.0078690.s001.docx]

**Appendix S1**

This appendix provides a step-by-step description of the procedure used to estimate natural direct and indirect effects (exemplified using alcohol consumption as the mediator). For a theoretical justification and description of the method see Lange *et al* [17]. To make the technique more readily available to other researchers and to facilitate reproducibility of our results, we also provide the used computer code (written in the statistical software ‘R’ programming language).

The first step involved fitting a model of the mediator (alcohol consumption) conditioning on exposure (SEP) and confounders of the exposure-mediator relation (age at baseline and study origin). Since the mediator, alcohol consumption, has three levels (<1, 1–7, and >7 drinks/week), we fitted a multinomial logistic regression model conditioning on SEP, age at baseline and study origin.

In a later step, the values of SEP were changed in order to do predictions, therefore a copy of the SEP variable was employed (‘SEPcopy’):

**STEP 1**

mydata$SEPcopy <- mydata$SEP

library(VGAM)

fitM <- vglm(alcohol ~ factor(SEPcopy) + age + factor(study),

data = mydata, family = multinomial())

Next, copies of the ID variable and the exposure (SEPx) were constructed. The new variable ‘SEPx’ corresponds to the value of the exposure through the indirect path. The original data set was replicated three times to allow for SEPx to take the three different possible values:

**STEP 2**

N <- nrow(mydata)

mydata$ID <- 1:N

LevelsOfSEP <- unique(mydata$SEP)

mydata1 <- mydata

mydata2 <- mydata

mydata3 <- mydata

mydata1$SEPx <- LevelsOfSEP[1]

mydata2$SEPx <- LevelsOfSEP[2]

mydata3$SEPx <- LevelsOfSEP[3]

newMyData <- rbind(mydata1, mydata2, mydata3)

The third step consisted of:

1. Calculation the probability of obtaining the mediator actually obtained using first the actual exposure and then the newly constructed auxiliary exposure.
2. Computing the weight given to each row in the extended dataset by dividing the two probabilities obtained in a:

**STEP 3**

newMyData$SEPcopy <- newMyData$SEP

tempDIR <- as.matrix(predict(fitM, type = "response", newdata=newMyData))[cbind(1:(3*N), newMyData$Alcohol)]

newMyData$SEPcopy <- newMyData$SEPx

tempINDIR <- as.matrix(predict(fitM, type = "response",

newdata=newMyData))[cbind(1:(3*N), newMyData$Alcohol)]

newMyData$weightM <- tempINDIR/tempDIR

Finally the marginal structural model for the direct effect of socioeconomic position and the indirect effect of alcohol consumption was fitted using the weights computed in the last step:

**STEP 4**

library(timereg)

fitYaalen <- aalen(Surv(FU_BC, BRSTC) ~ const(factor(SEP)) + const(factor(SEPx)) + const(factor(study)) + age,

data=newMyData, weights=newMyData$weightM, clusters=newMyData$ID)

summary(fitYaalen)

The direct and indirect effects and standard errors were derived directly from this output summary. The total effect was obtained by the sum of the two separate effects.

Confidence intervals for total effects and mediated proportions were computed by simulation using the following procedure:

**STEP 5**

## define function – the argument v should be vector indicating which coefficients to add to

## obtain the total effect

getTE <- function(fitAalen, v)

{

  TE <- sum(fitAalen$gamma[v])

  mu <- fitAalen$gamma[v]

  Omega <- fitAalen$robvar.gamma[v,v]

  require(MASS)

  temp <- mvrnorm(n=10^4, mu=mu, Sigma=Omega)

  temp_TE <- apply(temp,1,sum)

  med_prop <- c(mu/TE,1)

  med_prop_CI <- rbind(t(apply(temp/temp_TE, 2, quantile, c(0.025, 0.975))), c(1,1))

  output <- cbind(c(mu,TE), c(apply(temp,2,sd),sd(temp_TE)), med_prop, med_prop_CI)

  colnames(output) <- c("Est.", "SE", "med_prop", "lowerCI", "UpperCI")

  rownames(output) <- c(rownames(fitAalen$gamma)[v],"TE")

  return(output)

}

getTE(fitAalen, c(1,3))

getTE(fitAalen, c(2,4))
